# Histopathological distinction of non-invasive and invasive bladder cancers using machine learning approaches

**DOI:** 10.1186/s12911-020-01185-z

**Published:** 2020-07-17

**Authors:** Peng-Nien Yin, Kishan KC, Shishi Wei, Qi Yu, Rui Li, Anne R. Haake, Hiroshi Miyamoto, Feng Cui

**Affiliations:** 1 Thomas H. Gosnell School of Life Sciences, Rochester Institute of Technology, 1 Lomb Memorial Drive, Rochester, NY 14623 USA; 2 Golisano College of Computing and Information Sciences, Rochester Institute of Technology, 20 Lomb Memorial Drive, Rochester, NY 14623 USA; 3 Department of Pathology and Laboratory Medicine, University of Rochester Medical Center, 601 Elmwood Avenue, Rochester, NY 14642 USA

**Keywords:** Machine learning, Deep learning, Bladder cancer, Histopathology images

## Abstract

**Background:**

One of the most challenging tasks for bladder cancer diagnosis is to histologically differentiate two early stages, non-invasive Ta and superficially invasive T1, the latter of which is associated with a significantly higher risk of disease progression. Indeed, in a considerable number of cases, Ta and T1 tumors look very similar under microscope, making the distinction very difficult even for experienced pathologists. Thus, there is an urgent need for a favoring system based on machine learning (ML) to distinguish between the two stages of bladder cancer.

**Methods:**

A total of 1177 images of bladder tumor tissues stained by hematoxylin and eosin were collected by pathologists at University of Rochester Medical Center, which included 460 non-invasive (stage Ta) and 717 invasive (stage T1) tumors. Automatic pipelines were developed to extract features for three invasive patterns characteristic to the T1 stage bladder cancer (i.e., desmoplastic reaction, retraction artifact, and abundant pinker cytoplasm), using imaging processing software ImageJ and CellProfiler. Features extracted from the images were analyzed by a suite of machine learning approaches.

**Results:**

We extracted nearly 700 features from the Ta and T1 tumor images. Unsupervised clustering analysis failed to distinguish hematoxylin and eosin images of Ta vs. T1 tumors. With a reduced set of features, we successfully distinguished 1177 Ta or T1 images with an accuracy of 91–96% by six supervised learning methods. By contrast, convolutional neural network (CNN) models that automatically extract features from images produced an accuracy of 84%, indicating that feature extraction driven by domain knowledge outperforms CNN-based automatic feature extraction. Further analysis revealed that desmoplastic reaction was more important than the other two patterns, and the number and size of nuclei of tumor cells were the most predictive features.

**Conclusions:**

We provide a ML-empowered, feature-centered, and interpretable diagnostic system to facilitate the accurate staging of Ta and T1 diseases, which has a potential to apply to other types of cancer.

## Background

Bladder cancer is one of the most common malignancies in the world, with nearly 550,000 newly diagnosed cases and 200,000 deaths of this disease estimated in 2018 [1]. Approximately 90% of bladder cancers are urothelial carcinomas that arise from epithelial cells lining the inside of the bladder. Roughly three-fourths of urothelial carcinomas are non-muscle invasive [2]. According to the current WHO classification system, non-muscle invasive bladder cancers (NMIBCs) can be divided into three groups: Ta (non-invasive papillary), Tis (carcinoma in situ), and T1 (invasion into subepithelial connective tissue/lamina propria), which account for approximately 70, 10, and 20% of NMIBC, respectively [2, 3]. Ta and Tis tumors are confined to the urothelium and have not penetrated the basal membrane. In particular, Ta tumors often present as low-grade lesions that can often be managed conservatively [2–4]. By contrast, T1 tumors are mostly high-grade and have the potential to progress to muscle invasion and extravesical dissemination [3, 5]. In general, NMIBCs have a favorable treatment outcome with the five-year survival rate up to 90%, whereas muscle-invasive bladder cancers have a less favorable prognosis with 30–70% five-year survival rate [6]. Clearly, accurate diagnosis of non-invasive (Ta) versus invasive (T1) bladder cancers is vitally important and will help clinicians to make a timely and appropriate treatment plan for patients.

To date, the detection of bladder cancers mainly depends on the cystoscopic examination of the bladder and biopsy/resection of the tumor as well as urine cytology [7]. Currently, no molecular biomarkers accurately stage Ta and T1 tumors. Histological assessment remains a vital tool to differentiate the T1 disease from the Ta disease.

Although several histological features suggestive of tumor invasion have been identified (see below), Ta and T1 tumors sometimes look very similar under microscope, making the distinction very difficult even for experienced pathologists. As an illustration, 235 bladder tumors initially diagnosed as T1 tumors were restaged as being Ta (35%), T1 (56%), “at least” T1 (6%), and ≥ T2 (3%) diseases by an experienced reviewer [8]. Obviously, there is considerable room for improvement of inter-observer agreement by developing objective methods.

Computerized image processing technology has been shown to improve efficiency, accuracy and consistency in histopathological slide evaluation and provides a novel diagnostic tool to the practice of pathology [9]. Automated analysis systems have been developed to quantitatively capture morphological features of histopathological images to predict the outcome of breast cancer [10], neuroblastoma [11], lymphoma [12], lung cancer [13], and Barrett’s esophagus [14].

Image-based predictive models are further empowered by recent advances in machine learning (ML) and computer vision to achieve expert-level accuracy in medical image classification [15–20]. Recent work has shown that a convolutional neural network (CNN)-based deep learning model can achieve 100% accuracy in identifying the presence or absence of breast cancer cells in a whole slide [20]. A similar study from Google Inc. found that a CNN model was able to identify breast cancer better than pathologists [21]. Because training a CNN model from scratch requires a large number of medical images that are often hard to obtain, a highly effective approach to deep learning on small image datasets is to use a pre-trained network such as Visual Geometry Group (VGG) [22], which has previously been trained on large image-classification datasets. However, none of these models are built for classifying bladder cancer images. This lack of computational models severely hampers the application of modern image-based analytic tools to differentiating Ta and T1 diseases.

In this study, we aim to develop a novel ML-empowered, feature-centered, and interpretable diagnostic system to facilitate the accurate staging of Ta and T1 bladder tumors. We design a fully automated informatics pipeline to extract quantitative image features from hematoxylin and eosin (H&E)-stained slides (see flowchart in Supplementary Figure S1) and identify microscopic patterns that are important for distinguishing T1 from Ta tumors. Our methods may be not only helpful for the precision medicine of bladder cancer but also extensible to other types of cancer.

## Methods

### Histopathological slides

Upon approval from the Institutional Review Board at University of Rochester Medical Center (URMC), we collected a total of 1177 images from H&E-stained bladder cancer tissues, which included 460 non-invasive (stage Ta) and 717 invasive (stage T1) urothelial tumors. Problematic cases where it was difficult for a group of genitourinary pathologists at URMC to histopathologically distinguish between Ta and T1, as well as muscle-invasive cases (stage T2 and above), were excluded from the analysis. All images were included for image processing and analysis, with the labels of images serving as ground truth. All tumor specimens were obtained by surgical excision and processed by a standardized protocol at the Department of Pathology and Laboratory Medicine at URMC.

### Image digitization system

A Leica upright microscope DM5000 B research microscope attached with a high-resolution camera from MacroFire was used to capture the raw H&E-stained images. The camera was able to capture a field of 2048 × 2048 pixels under the 100× magnification. Image files were saved in the “.tiff” format. The central part of the raw images was cropped into 1 to 4 images with 700 × 700 pixels by an ImageJ-based script with the “Crop” function in ImageJ.

### Image processing system

The cropped images were then pre-processed with the “FFT” function in ImageJ if needed because this function made the light intensity evenly distributed by normalizing the intensity from the darkest corner to the brightest corner on the image. These pre-processed images were then converted into black and white images to mask the irrelevant areas. The images with lesions of interest were used for feature extraction.

### Image feature extraction

The feature extraction was performed using CellProfiler and ImageJ. Both packages enabled us to create and customize pipelines for extracting patterns in the images. ImageJ provides a scripting language MacroJ, which allows the extraction of patterns of interest. In this project, the patterns of retraction artifact, nuclear size, and cytoplasmic color were extracted by ImageJ. CellProfiler provides multiple built-in cellular feature extraction modules. The patterns of connective tissue around the tumor and nuclear shapes in the images were extracted by CellProfiler. Overall, ImageJ extracted textural features in the whole tissue, whereas CellProfiler extracted features of individual cells. For every image, 60 features were extracted by ImageJ and 636 features were extracted by CellProfilers. The spreadsheets containing the features from ImageJ and CellProfiler were merged into a single data frame in R environment.

### Statistical analysis and plotting

All statistical analyses in the project were performed using R. To set the bin size for color spectrums, all image pixels were processed and evaluated through R scripts. The performance metrics including accuracy, receiver operating characteristic (ROC), and area under the curve (AUC) were calculated by the functions in the Scikit-Learn package. ROC and AUC are appropriate metrics because our data have imbalanced classes: 460 non-invasive and 717 invasive samples. The plots of ROC curves, cutoff points and AUC scores were generated by the Matplotlib package. The boxplots that compare the performance of ML models were generated by SigmaPlot 12.5.

### Data processing methods

Features extracted by ImageJ and CellProfiler were processed and the data were saved in the comma separated value (CSV) format. An R script was written to combine all CSV files and produce a large spreadsheet. The Pandas package was used for data processing and subsetting. The large CSV file was transformed into the Numpy matrix before putting into ML models.

### General ML models

Several general ML classifiers used in the project were taken from several Python packages. The probabilistic neural network framework was from the Neupy package. All of the ensemble learning models, including probabilistic neural network (PNN), support vector machine (SVM), logistic regression (LR), bagging (Adaboot), random forest (RF), and multilayer perceptron (MLP), were from the Scikit-Learn package. The datasets were randomly partitioned into a training set (70%) and a testing set (30%). To ensure the robustness of the results, the random partitioning process was repeated 20 times and the mean performance of the 20 tests was used to represent the overall performance of the classifier. The default threshold 0.5 was used for classification.

### Training and validation dataset splitting

Random sampling was performed multiple times in each training and validation. Before feeding the data into any machine learning models or deep learning models, the dataset was first balanced by sampling an equal number of 460 invasive cases and 460 non-invasive cases before the training started. The weight of invasive cases is equal to the weight of non-invasive cases. Then within the balanced dataset, data were further split into a 70% training set and a 30% validation set through random sampling. Not image or data appeared in both data set and no duplicates were allowed in the study.

## Results

### Distinctive microscopic patterns for stage ta versus T1 bladder tumors

**Fig. 1:**
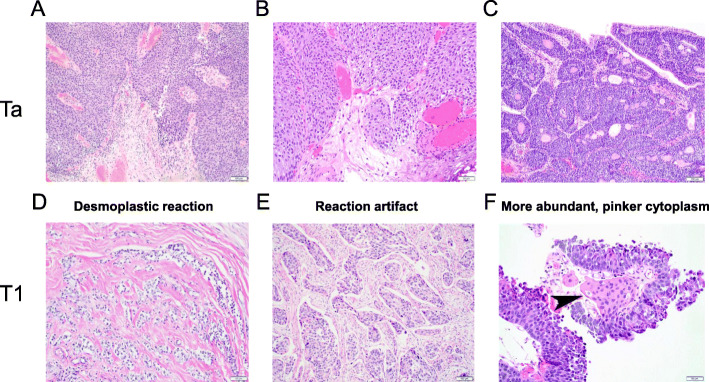
Histological features of stages Ta (**a-c**) versus T1 (**d-f**) bladder cancers. Three microscopic patterns, including desmoplastic reaction (**d**), retraction artifact (**e**), and more abundant, pinker cytoplasm (**f**, arrowhead), are apparent in invasive components of T1 tumors, but not in Ta tumors without (**a**, **b**) or with (**c**) an inverted growth pattern. Original magnification: **a**, **c**, **e** – 100x; **b**, **d**, **f** – 200x

At least three morphological features have been identified to distinguish between stages Ta (Fig. 1a-c) and T1 (Fig. 1d-f) bladder tumors. The first pattern is desmoplastic reaction, which is characterized with dense fibrosis around the nests of T1 tumor cells (Fig. 1d). This pattern is most definite for invasion, but T1 lesions often lack it. The second pattern is retraction artifact, which is the result of tissue shrinkage after dehydration during tissue processing, seen around the nests of T1 tumor cells (Fig. 1e). The third pattern is more abundant, pinker cytoplasm in T1 tumor cells, presumably due to higher uptake of eosin, compared with that of Ta tumor cells (Fig. 1f). Although pathologists usually make a diagnosis of tumor invasion based on these patterns under microscope, a quantitative representation will allow automatic extraction and analysis of the patterns in H&E-stained slides.

### H&E-stained slide digitalization, image processing and feature extraction

We obtained 1177 H&E-stained histopathology images of Ta or T1 bladder tumors from the archive in the Department of Pathology and Laboratory Medicine at URMC. To digitize these slides, each image was captured at × 100 magnification with 2048 × 2048 pixels. Although the overall images were very clear, a dark spot was often found at the lower right-hand corner. We therefore cropped and tiled the central part of the raw images to get smaller ones with 700 × 700 pixels.

To extract objective morphological information from these images, we used ImageJ and CellProfiler to extract image patterns into numerical numbers. We therefore built nine fully automated image pattern extraction pipelines to capture the above three microscopic patterns. Due to the complexity of pathological images, each pattern consisted of various features. The general procedure of feature extraction is described below. We first masked unwanted areas using methods like color thresholding and matrix subtractions before extracting the features. Since all of the raw images were consistent in staining quality, the parameters for extracting each feature were kept the same across all images. The image features included nuclear size distribution, crack edge, sample ratio, distribution of pixel intensity in the connective tissue and cytoplasm, as well as the shape of connective tissue and nuclei of tumor cells. The numerical representation of the features was outputted in spreadsheets and placed in columns.

**Fig. 2:**
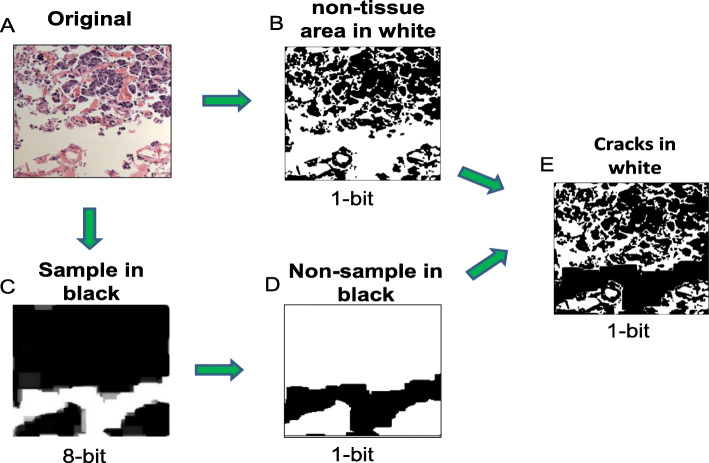
Flow diagram of the image processing method for extracting features from the retraction artifact pattern

For example, to extract the retraction artifact pattern, we developed a pipeline to differentiate two types of non-tissue regions in a H&E-stained image, one was around cells (i.e., small space around cells) and the other was between tissue parts (i.e., large space between tissue edges) (Fig. 2a). To only catch the small space surrounding cells (named “cracks” for simplicity), we first converted an original color image to a monochrome image with black or white color on each pixel and all non-tissue regions were in white (Fig. 2b). Then we converted the original color image to an 8-bit grayscale image. The regions with more than 40 pixels in diameters were considered to be the regions between tissue parts, which were shown in white (Fig. 2c). This 8-bit image was then converted to a 1-bit image with black and white colors inverted; now the inter-tissue space was shown in black (Fig. 2d). Then we combined images shown in Fig. 2b and d to get the final image in which the inter-tissue spacer was masked and cracks were shown in white (Fig. 2e). The number of pixels in white regions represented the size of cracks around cells.

We also developed pipelines (Supplementary Figures S2 and S3) to extract features in the pinker cytoplasm pattern and the desmoplastic reaction pattern. Note that each of the three microscopic patterns was extracted separately, and the numeric representation of each pattern was later combined into a large spreadsheet in the CSV format, in which each row represented an image and each column represented a feature. For every image (out of 1177), 740 quantitative features were extracted to represent the three microscopic patterns.

### Unsupervised clustering of cancer images

**Fig. 3:**
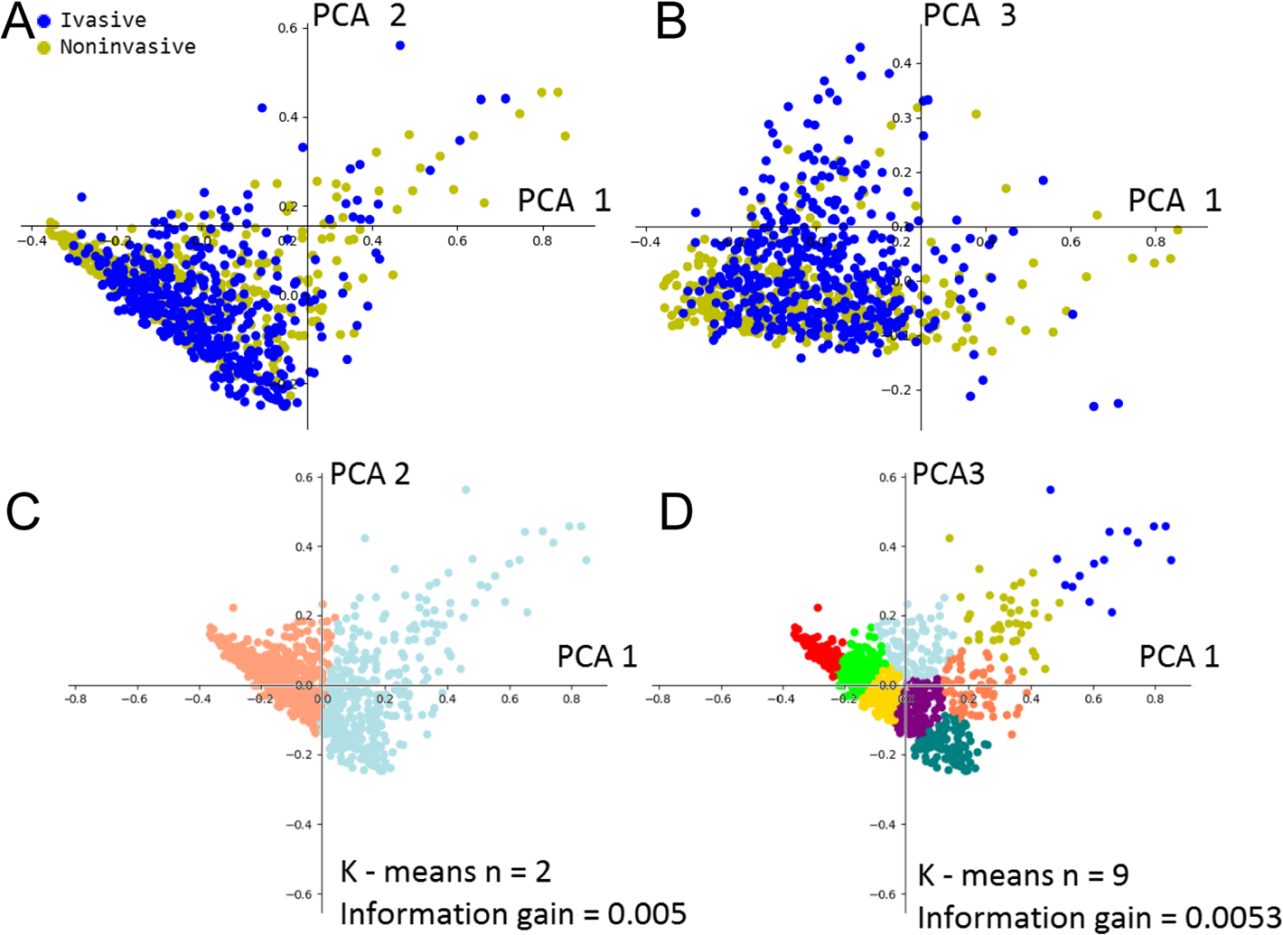
Clustering analysis of extracted features from Ta (non-invasive) and T1 (invasive) tumor images. The features were first selected by PCA and then clustered using k-means analysis. Plots were made for the first and second components of PCA output (**a**), which were clustered with k = 2 (**c**). Plots were also made for the first and third components of PCA output (**b**), which were clustered with k = 9 (**d**)

To understand whether extracted features were sufficient to differentiate the histopathological images of Ta and T1 tumors, we set out to conduct a cluster analysis of the features. We first reduced data dimension through principal component analysis (PCA) because, through PCA, we were able to rank top components by their eigenvalues. However, as shown in Fig. 3a-b, plotting the top components with the highest eigenvalue failed to find recognizable clusters. In addition, by performing k-means analysis on PCA components, we found no apparent clusters between k = 2 and k = 9 (Fig. 3c-d). Combining the PCA and k-means analyses, we found that the non-invasive and invasive tumor images were highly overlapped. Splitting the clusters resulted in less than 0.006 in information gain. These data suggested that non-invasive and invasive bladder cancer images were not separable with simple linear transformation. Therefore, supervised learning methods were considered.

### Feature reduction and supervised classification of cancer images

To select meaningful ones from the 740 features extracted by ImageJ and CellProfilers, we first manually trimmed questionable features that were related to time (i.e., time when images are processed or taken), index (i.e., labels of images), descriptive string (i.e., initials of processing methods or channels of image processing), or those containing missing values ‘N/A’ as the results of ImageJ and CellProfilers processing. In addition, the features containing no numeric values were also removed. As a result, 696 features were selected for further analysis.

**Fig. 4:**
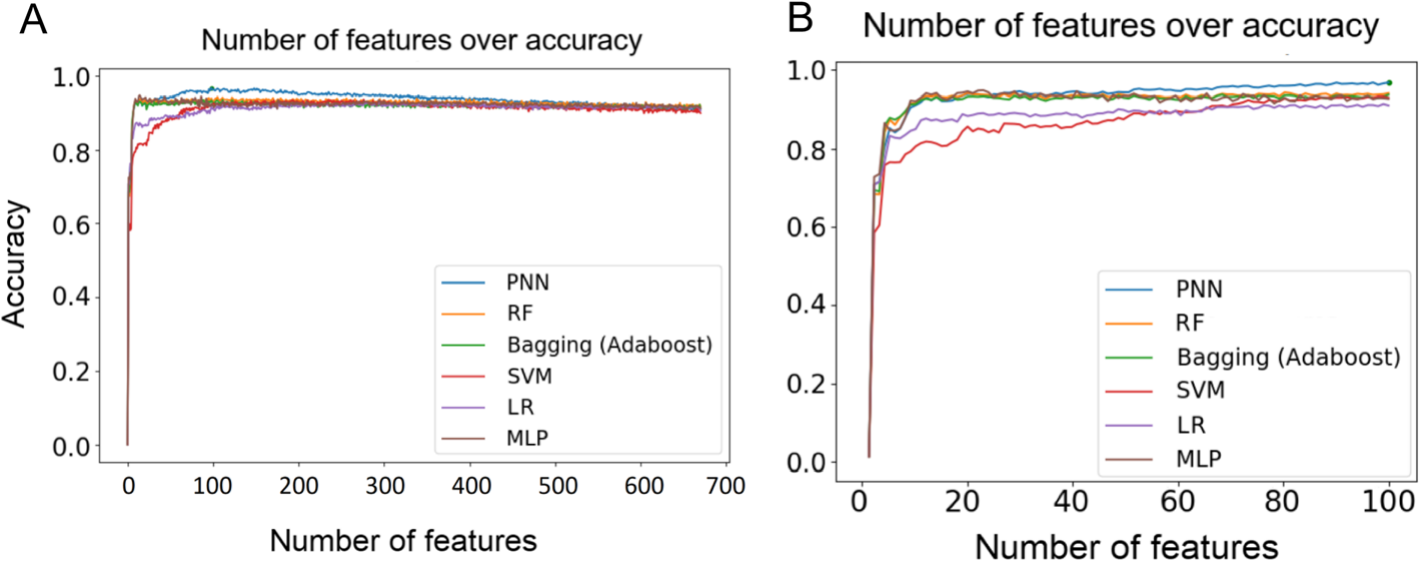
Prediction accuracy of 6 ML models for 696 features (**a**) and top 100 features (**b**). The importance of 696 features was ranked by decision trees. The features were added to the models in the order of the importance

Given that the training set contained 930 images, 696 features might raise the concern of overfitting. To address this concern, we reduced the number of features by employing decision tree (DT) with k-fold cross-validation to rank the relative importance of the features. Specifically, we first used all 696 features as the input to build 20 forests, each with 40 trees. Similar to RF, each tree was constructed by random samples, but the number of features was fixed to 696. The DT method was used to evaluate the importance of each feature by averaging the importance values of the feature in all trees of a forest. We therefore ranked the relative importance of all 696 features based on their average importance values. This rank determined the order of the features added to ML models. That is, after measuring the impact of the first feature, we iteratively added the next feature in the rank to the models. As shown in Fig. 4a, as the features were added in the ranking order, the performance of 6 ML classifiers including PNN, increased and reached a plateau between 70th and 100th features (Fig. 4b). After adding 200 features, the performance started to drop (Fig. 4a). To examine whether the ranking order of features was critical for the observed tendency, we randomized the feature order and found that a plateau was still reached between 70th and 100th features (Supplementary Figure S4A-B). This result suggests that the DT method successfully selects the most important 100 features from the original 696 features.

**Fig. 5:**
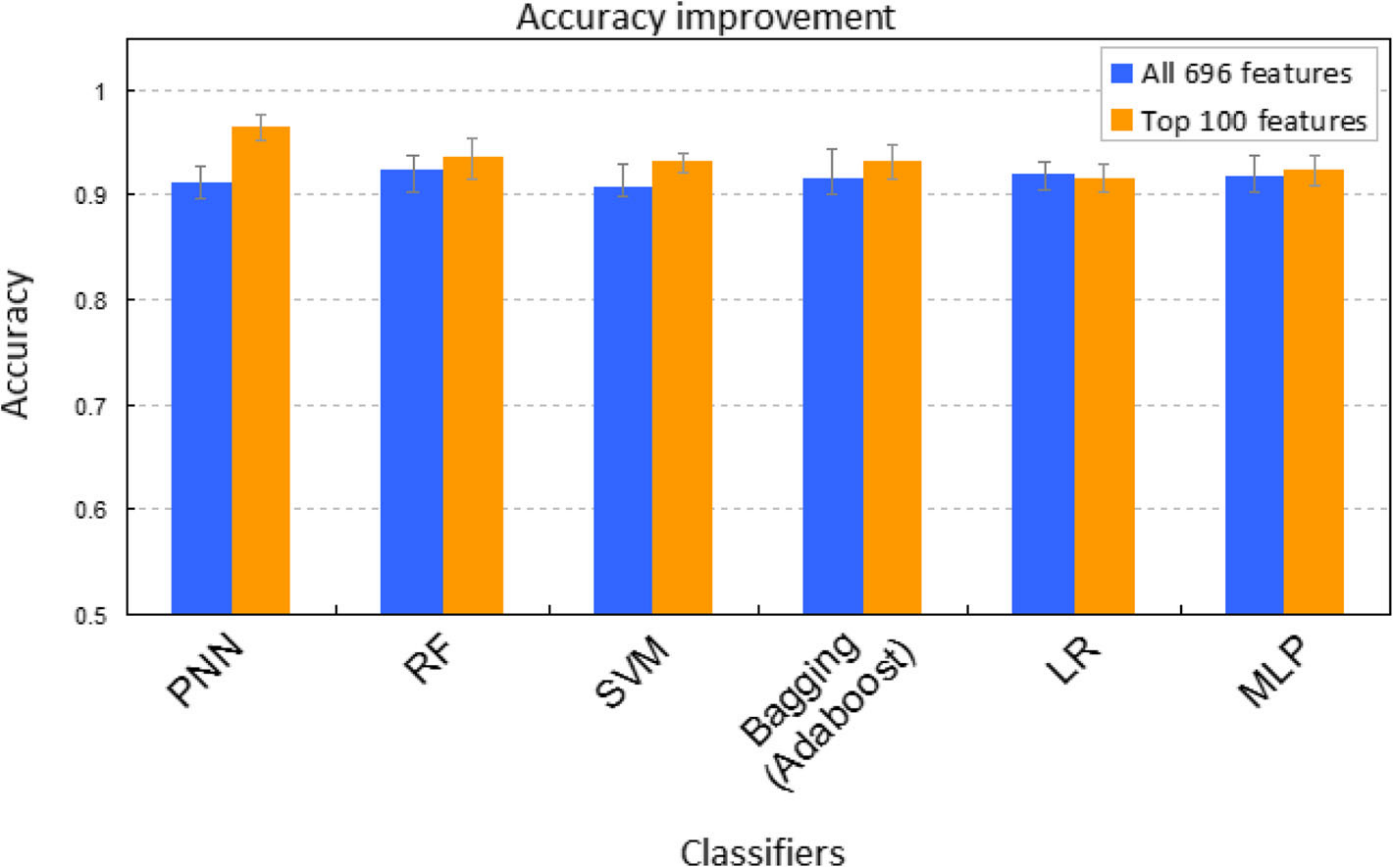
Accuracy comparison for 6 ML methods based on 696 features (blue) and top 100 features (orange)

**Fig. 6:**
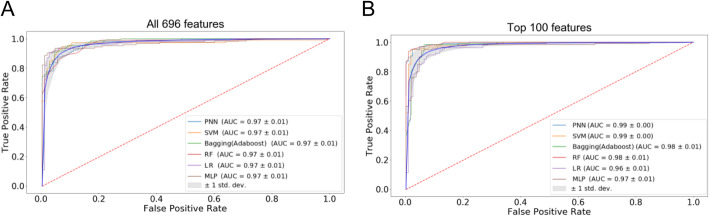
ROC curves of 6 ML classifiers based on all 696 features (**a**) and top 100 features (**b**). All 696 features or the top 100 features evaluated by multiple decision trees were incorporated into 6 ML classifiers to calculate the ROC curves. AUC values of the models were indicated in the caption. Twenty iterations were performed for each model. The mean ROC curve (blue) and standard deviation (grey shade area) are presented

To compare further the two feature sets (100 vs. 696) in predicting Ta and T1 bladder cancers, we used 6 ML classifiers, including PNN [23–25], RF [26, 27], SVM [28], bagging (Adaboost) [29], LR [30], and MLP. Three metrics were used to evaluate the performance of the classifiers, including accuracy, ROC curve, and the AUC. We found that the average accuracy was over 90% for all classifiers (Fig. 5). Moreover, the 100-feature set outperformed the 694-feature set in five out of six classifiers, except LR (Fig. 5). The same trend is observed in ROC and AUC (Fig. 6a-b and Supplementary Figure S5A-B). Of note, PNN outperformed other classifiers with the AUC of 0.99 (Fig. 6b and Supplementary Figure S5B) and the accuracy of 96.7% (Fig. 5). Overall, our work clearly showed that the top 100 features generally had a higher predictive power than the 696 features.

**Fig. 7:**
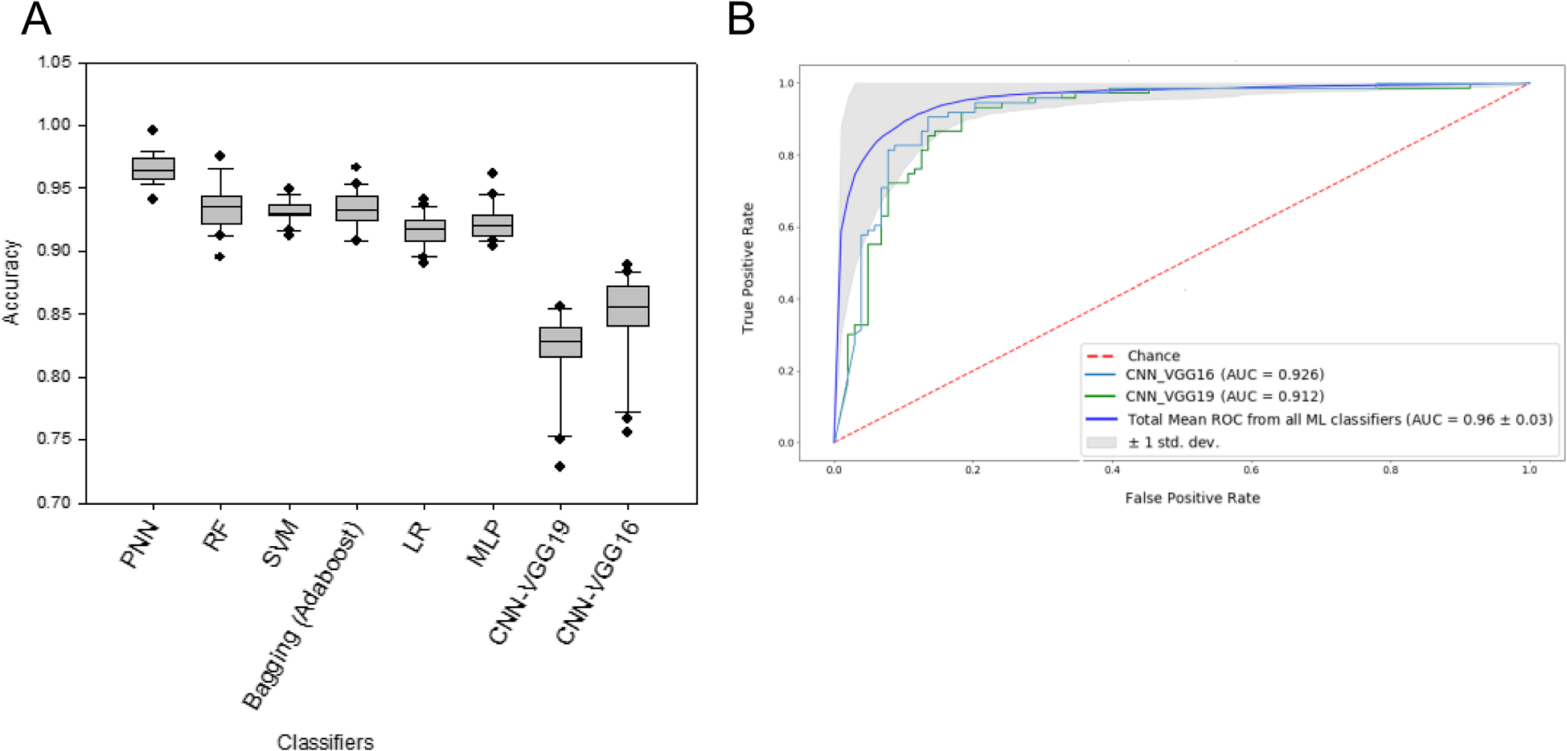
Prediction accuracy and ROC curves of CNN-based models. Pre-trained VGG16 and VGG19 networks were used to build the models. AUC values of the models were indicated in the caption. Twenty iterations were performed for each model. The mean ROC curve (blue) and standard deviation (grey shade area) are presented

To examine the performance of deep learning models on our data, we used both pre-trained VGG16 and VGG19 networks to extract features. Specifically, we took the convolutional base of the networks, ran the Ta and T1 cancer images through it, and trained a new classifier on top of the output. We found that the accuracies of VGG16 and VGG19 reached 84 and 81%, respectively (Fig. 7a and Supplementary Figure S6A), whereas their AUC values were 0.926 and 0.912, respectively (Fig. 7b and Supplementary Figure S6B). Our results showed that the general ML classifiers outperformed deep learning models, suggesting that, for cancer histopathological images, feature extraction based on domain knowledge performed better than computer-based feature extraction.

### Relative importance of three microscopic patterns

**Fig. 8:**
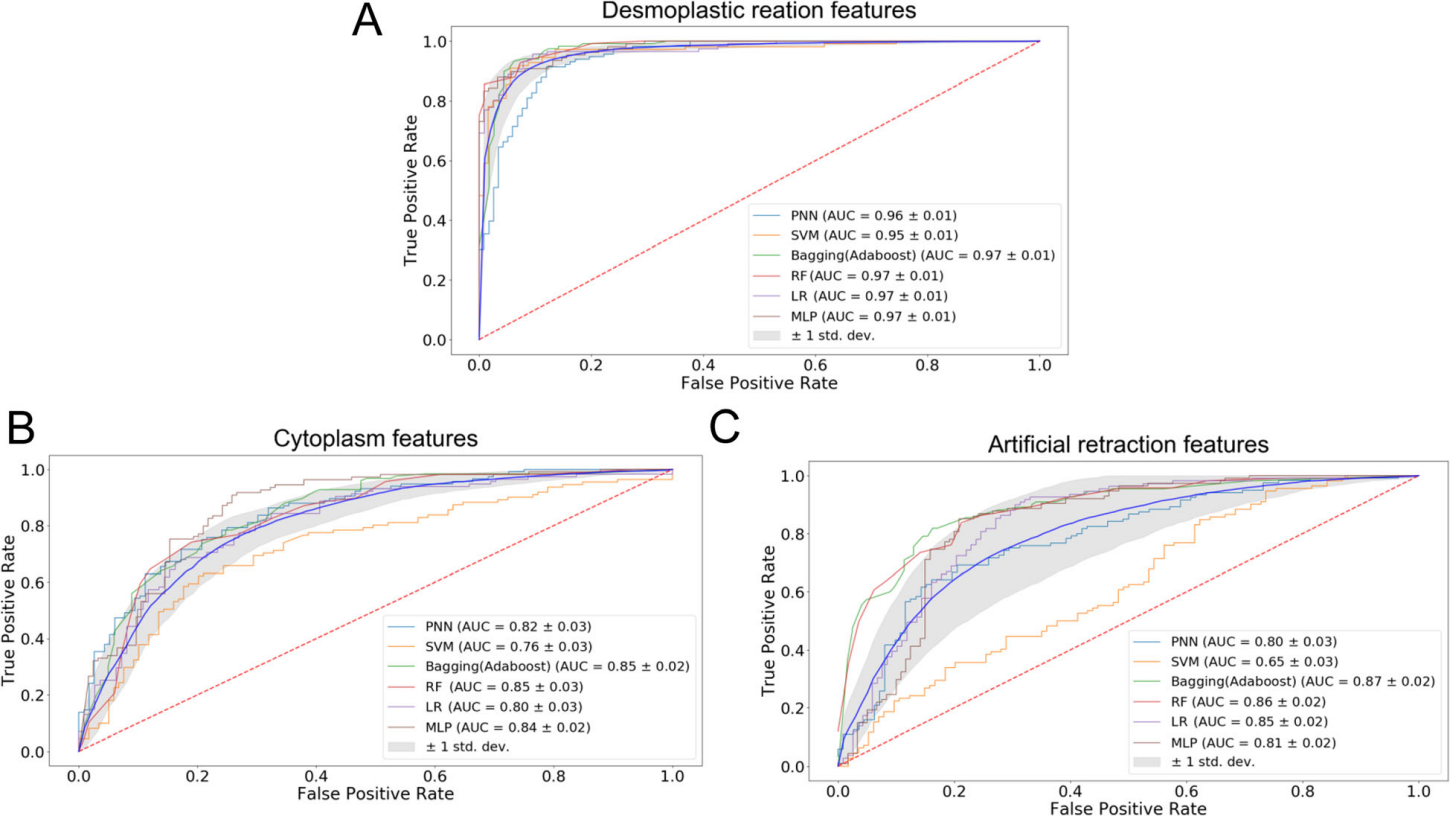
ROC curve of various ML classifiers based on features related to desmoplastic reaction (**a**), cytoplasmic/eosin intensity (**b**), and retraction artifact (**c**). There are 675, 13, and 15 features used to represent the desmoplastic reaction, cytoplasmic/eosin intensity and retraction artifact patterns, respectively. Several ‘background’ features were shared by the three patterns, leading to the sum of the features (i.e., 703) larger than the total number of features (i.e., 696). The features were incorporated into 6 ML classifiers to calculate the ROC curves. AUC values of the models were indicated in the caption. Twenty iterations were performed for each model. The mean ROC curve (blue) and standard deviation (grey shade area) are presented

To assess the relative importance of the three microscopic patterns in predicting non-invasive versus invasive bladder cancer images, we separated the 696 features into three groups and assessed the performance of the 6 ML classifiers. We found that features extracted from the desmoplastic reaction pattern had the highest overall accuracy of 90.5% with the average AUC values of 0.98 (Fig. 8a and Supplementary Figure S7A). By contrast, pinker cytoplasm had 74.5% overall accuracy with the average AUC of 0.825 (Fig. 8b and Supplementary Figure S7B), whereas retraction artifact had 73.4% overall accuracy with the average AUC values of 0.802 (Fig. 8c and Supplementary Figure S7C). It was noteworthy that desmoplastic reaction had 675 features, whereas pinker cytoplasm and retraction artifact had 13 and 15 features, respectively. These observations suggest that the models with the desmoplastic reaction features may be overfitting. Reducing from 675 features to 70 features in the desmoplastic reaction pattern still outperformed the pinker cytoplasm and retraction artifact patterns with an accuracy of over 90% (data not shown). To some extent, all three patterns could distinguish Ta and T1 tumor images with a reasonable accuracy (> 70%), suggesting that some features extracted from these patterns might be correlated (see Discussion).

To understand which features in the desmoplastic reaction pattern are more important, we ranked all features based on 40 DTs. We found that features, such as the number of nuclei and distributions of nuclei sizes, came out at the very top of our ranking (Supplementary Figure S8). These findings were consistent with the main microscopic characteristics of the desmoplastic reaction pattern, in which a large number of inflammatory cells surround the nests of tumor cells. Our result suggests that the desmoplastic reaction pattern contains the most informative features in distinguishing Ta versus T1 bladder tumors.

## Discussion

The goal of this project was to build a ML-empowered, feature-centered, and interpretable diagnostic system to assist pathologists to distinguish histopathological images of non-invasive and invasive bladder cancers. For a given image, the system provided a probability value to Ta or T1 tumors, which can be used as additional evidence to facilitate the doctors’ decision-making process.

To this goal, we successfully developed automatic pipelines to extract features in three invasive patterns characteristic to the T1 stage bladder cancer (i.e., desmoplastic reaction, retraction artifact, and abundant pinker cytoplasm), using ImageJ and CellProfiler. Meanwhile, the presence of the muscle layer in the specimens of bladder tumor resection is often crucial for cancer staging. However, we have not taken into account the muscle layer in our analysis because: 1) its presence in a slide provides no help in distinguishing Ta and T1 tumors; and 2) the muscle layer is often absent in biopsy specimens. Our system was therefore designed on the basis of the assumption that the muscle layer is not present in tumor specimens. The fact that the system is able to achieve > 90% predictive accuracy suggests that textural features hidden in the aforementioned three patterns are critical for distinguishing T1 from Ta tumors.

We further investigated the relative importance of the three patterns in the distinction of Ta versus T1 tumors, and found that the desmoplastic reaction pattern is most important. Interestingly, using 15 and 13 features identified from retraction artifact and abundant pinker cytoplasm patterns respectively still achieve > 70% accuracy. A separate analysis with 60 features combining all features extracted from the retraction artifact pattern and the pinker cytoplasm pattern was able to achieve ~ 85% accuracy (data not shown). In our view, this high predictive accuracy may be explained by two possibilities. First, multiple patterns may co-exist in the T1 tumor images. In other words, most of T1 images may have more than one microscopic pattern. Second, different patterns may share common textural features. Identification of these ‘basic’ features will shed light on the fundamental differences between Ta and T1 tumors. It may help further reduce the feature number, thereby improving the interpretability of this ML-based diagnostic system.

Feature engineering requires domain knowledge/expertise and may take much time to identify features that represent the patterns of interest. Recently, deep learning techniques [31, 32] from the computer science field have dramatically improved the ability of computers to recognize objects in images. This raises the possibility for fully automated computer-aided diagnosis in pathology. Among all the ML models in image recognition, CNN is one of the most studied and validated method. Not only it has great performance, but also the design of CNN hidden layers allows the model to extract meaningful features without any prior knowledge. The pathology community has been showing increasing interests in comparing CNN to human judgements. Although applying the deep neural network to recognizing medical image patterns is not a new idea and has shown promising results, its requirement of large quantity of data for training turns out to be a big bottleneck for many unpopular diseases. To address this limitation, we developed CNN models using pre-trained VGG networks and found that it achieves a remarkable accuracy of 84%. Of note, these CNN models are pre-trained on general images that are different from histopathological images, suggesting that their performance could be improved with pre-training on histopathological images.

Notably, features in CNN models are automatically extracted from images without prior knowledge, and some features may be completely novel to pathologists. By assessing the intermediate layers of CNN, we may identify novel features that could be subsequently added to the feature engineering models to improve prediction accuracy. This iterative process will help make our system more powerful and interpretable.

Although the inclusion of pathologists (i.e., human-in-the-loop) in the model development process is very important, there is a need to go beyond interpretable machine learning. To reach a level supporting the pathologists in their daily decision making, another factor that should be taken into account is causability [33], which is measured in terms of effectiveness, efficiency, satisfaction related to causal understanding and its transparency for a user. In other words, it refers to a human understandable model. Since causability encompasses measurements for the quality of explanations, causability enables an expert pathologist to consider the causality of a particular disease. As such, although our system, in some sense, is interpretable, achieving causability is the ultimate goal of our system, which will be not only usable but also useful for pathologists.

## Conclusions

With ImageJ [34, 35] and CellProfiler [36], nearly 700 numeric features were extracted from three well-characterized patterns that distinguish T1 from Ta tumors, including desmoplastic reaction, retraction artifact, and abundant pinker cytoplasm. Clustering analysis with k-means failed to separate Ta and T1 images. To avoid overfitting, we selected only informative feature through feature ranking based on decision-trees with k-fold cross-validation. With the top 100 features, we successfully distinguished ~ 1200 Ta and T1 images with an accuracy of 91–96% using six classic ML approaches such as random forest, LR, PNN, bagging (Adaboost), SVM, and MLP. By contrast, a CNN model based on pre-trained VGG networks achieved an accuracy of 84%, suggesting that human-assisted feature extraction could outperform automatic feature extraction. Our analysis suggests that desmoplastic reaction is more important than the other two patterns. Moreover, the number and size distribution of nuclei of tumor cells in the desmoplastic reaction pattern appear to be the most predictive features, which is generally consistent with observations by pathologists. This ML-empowered diagnostic system is highly interpretable and has a potential to apply to other types of cancer.

## Supplementary information

**Additional file 1.**

## Data Availability

Example images, ImageJ macros, processing pipeline are available to interested readers at https://github.com/bennyyin/ImagejOnBladderH-E/branches. Anonymous datasets from the present study are available from the corresponding authors on reasonable requests. No identifying/confidential patient data was collected.

## Abbreviations

AUC: Area under the curve
CNN: Convolutional neural network
CSV: Comma separated value
DT: Decision tree
H&E: Hematoxylin and eosin
LR: Logistic regression
ML: Machine learning
MLP: Multilayer perceptron
NMIBC: Non-muscle invasive bladder cancer
PCA: Principal component analysis
ROC: Receiver operating characteristic
SVM: Support vector machine
VGG: Visual Geometry Group
