# Supplementary material for: Histopathological distinction of non-invasive and invasive bladder cancers using machine learning approaches
Source: BMC Med Inform Decis Mak. 2020 Jul 17;20:162. doi: 10.1186/s12911-020-01185-z (PMC7367328; doi:10.1186/s12911-020-01185-z)

**Supplementary Materials**

**Histopathological distinction of non-invasive and invasive bladder cancers using machine learning approaches**

Peng-Nien Yin^1^, Kishan Kc^2^, Shishi Wei^2^, Qi Yu^2^, Rui Li^2^, Anne R. Haake^2^, Hiroshi Miyamoto^3*^, and Feng Cui^1*^

^1^Thomas H. Gosnell School of Life Sciences, Rochester Institute of Technology, 1 Lomb Memorial Drive, Rochester, NY 14623, USA.

^2^Golisano College of Computing and Information Sciences, Rochester Institute of Technology, 20 Lomb Memorial Drive, Rochester, NY 14623, USA.

^3^Department of Pathology and Laboratory Medicine, University of Rochester Medical Center, 601 Elmwood Avenue, Rochester, NY 14642, USA.

Contents

**Supplementary Figures** . . . . . . . . . . . . . . . . . . . . . . . . . . . . . . . . . . . . . . . . . . . . . . . . . . . . . . . . . . . . . . 2

Figure S1 . . . . . . . . . . . . . . . . . . . . . . . . . . . . . . . . . . . . . . . . . . . . . . . . . . . . . . . . . . . . . . . . . . . . . . 2

Figure S2 . . . . . . . . . . . . . . . . . . . . . . . . . . . . . . . . . . . . . . . . . . . . . . . . . . . . . . . . . . . . . . . . . . . . . . 3

Figure S3 . . . . . . . . . . . . . . . . . . . . . . . . . . . . . . . . . . . . . . . . . . . . . . . . . . . . . . . . . . . . . . . . . . . . . . 4

Figure S4 . . . . . . . . . . . . . . . . . . . . . . . . . . . . . . . . . . . . . . . . . . . . . . . . . . . . . . . . . . . . . . . . . . . . . . 5

Figure S5 . . . . . . . . . . . . . . . . . . . . . . . . . . . . . . . . . . . . . . . . . . . . . . . . . . . . . . . . . . . . . . . . . . . . . . 6

Figure S6 . . . . . . . . . . . . . . . . . . . . . . . . . . . . . . . . . . . . . . . . . . . . . . . . . . . . . . . . . . . . . . . . . . . . . . 7

Figure S7 . . . . . . . . . . . . . . . . . . . . . . . . . . . . . . . . . . . . . . . . . . . . . . . . . . . . . . . . . . . . . . . . . . . . . . 8

Figure S8 . . . . . . . . . . . . . . . . . . . . . . . . . . . . . . . . . . . . . . . . . . . . . . . . . . . . . . . . . . . . . . . . . . . . . . 10


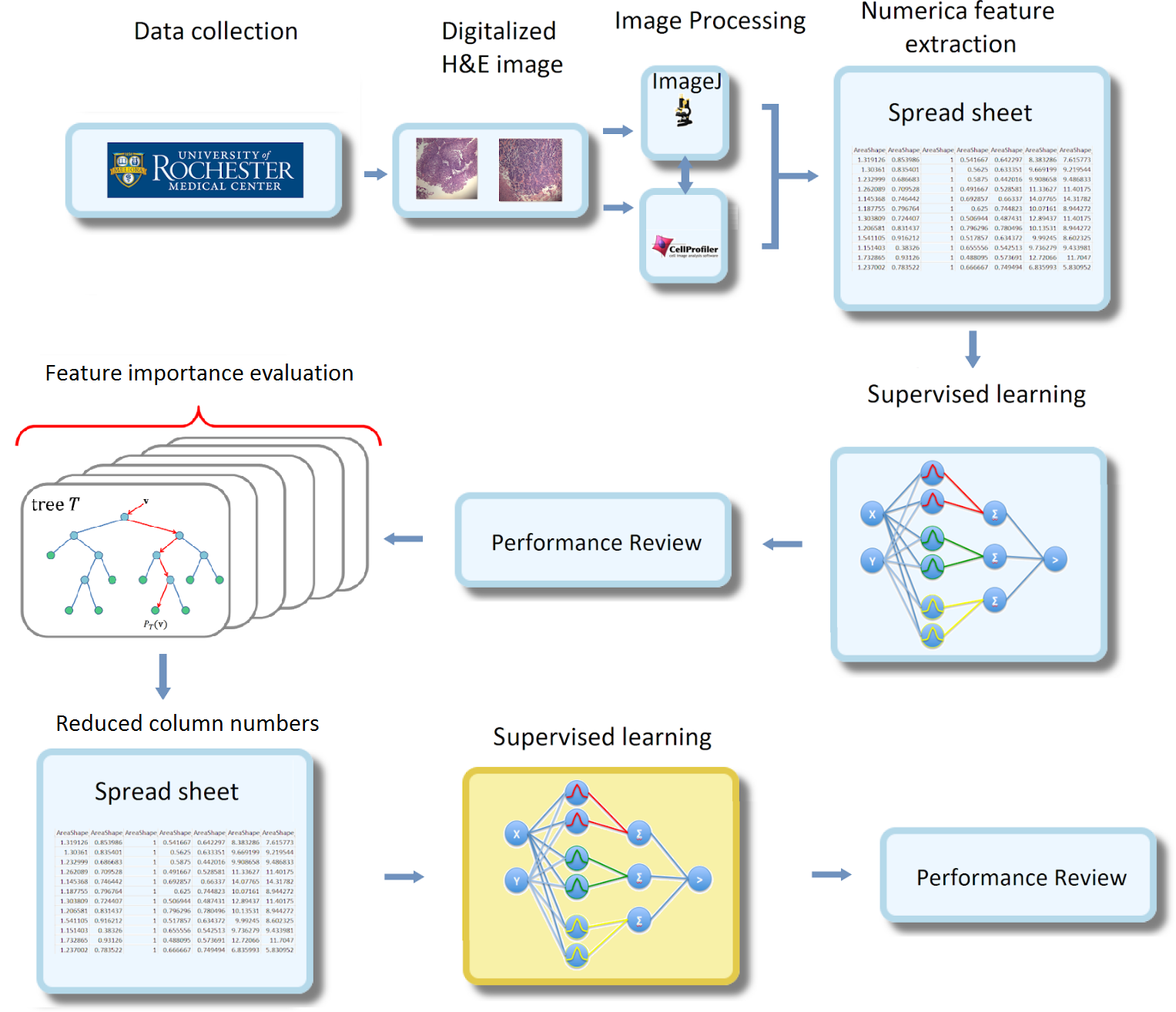


Figure S1: Flow chart for differentiating histopathological images of non-invasive and invasive bladder cancers using ML approaches. Images collected from the Department of Pathology and Laboratory Medicine at URMC were preprocessed by ImageJ and CellProfiler to extract features that are subsequently outputted as spreadsheets. Features were used to build various ML classifiers and the prediction performance was evaluated. The features were then ranked by their importance and a small set of important features was selected to build classifiers to improve the performance.

Figure S2: Flow diagram of the proposed image processing method for finding eosin intensity within the cytoplasm. The original color image (A) was converted to a 1-bit image by adjusting the color threshold so that the non-cytoplasmic regions are shown in white (B). At the same time, the eosin frame was extracted from the original color image through the color deconvolution method in ImageJ (C). The eosin frame was converted to an 8-bit grayscale image (D). Then two frames were merged through a matrix subtraction between the 8-bit eosin intensity image (D) and 1-bit image (B) to derive an image with eosin intensity within cytoplasm (E). The unwanted regions are shown in black (with value = 0) and the number of pixels with non-zero values represents the tissue areas with eosin uptake (F). All image processing was done in ImageJ.

Figure S3: An imaging processing procedure of how the desmoplastic reaction pattern was captured. The images were preprocessed to exclude unwanted regions including cell nuclei and red blood cells (A). The CNT regions were later segregated into different chunks and consider as different objects (B). Ultimately, features of each object, such as the shape and intensity, were captured and recorded as numeric values (C).

Figure S4: Prediction accuracy over the top 100 features in which each feature is added to the models by rank (A) or by a random order (B). The rank of the features was determined by its performances using random forest. The accuracy of multiple ML models was recorded each time before the next feature was added to the models based on a random order or an order previously the ranked by random forest.

A.


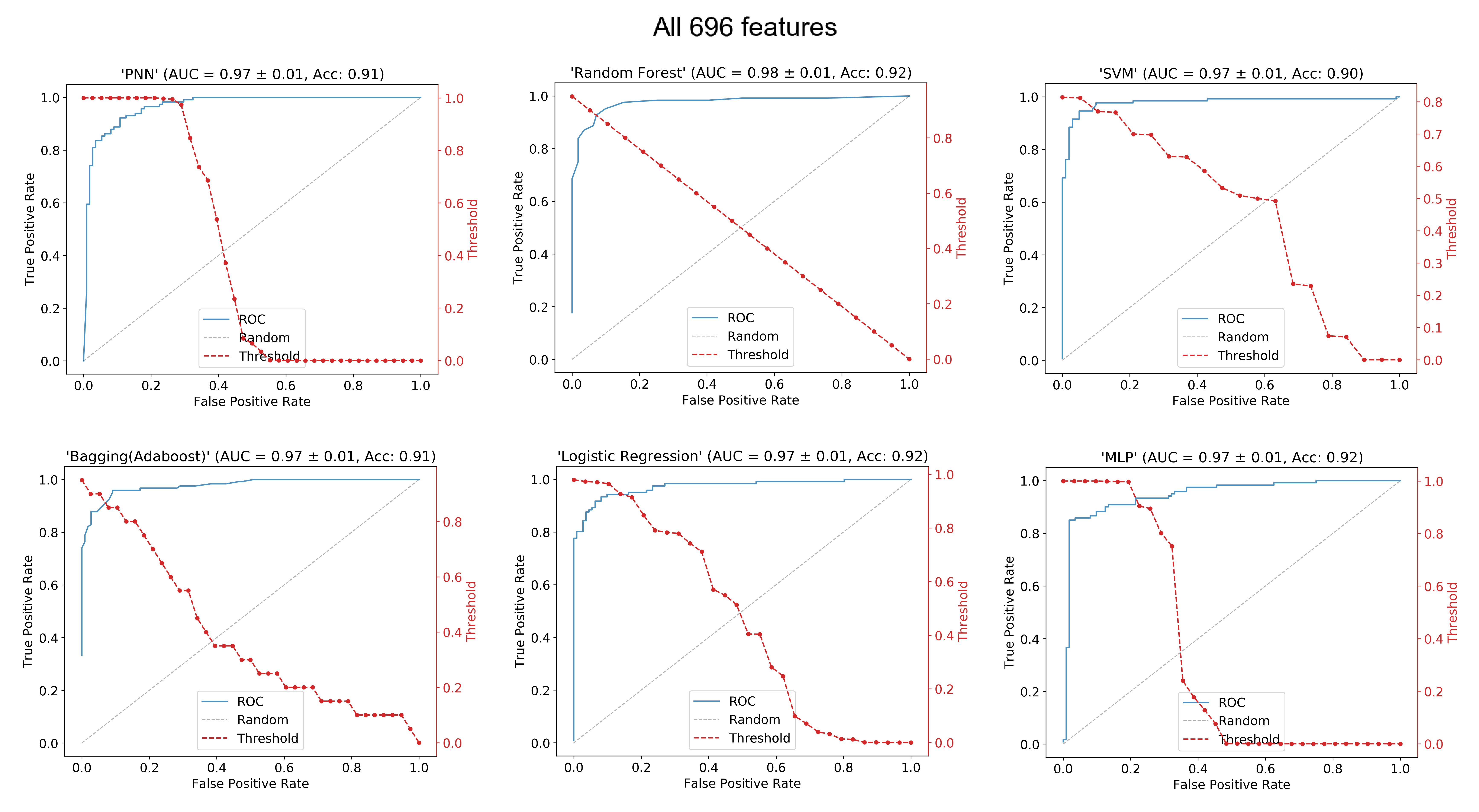


B.


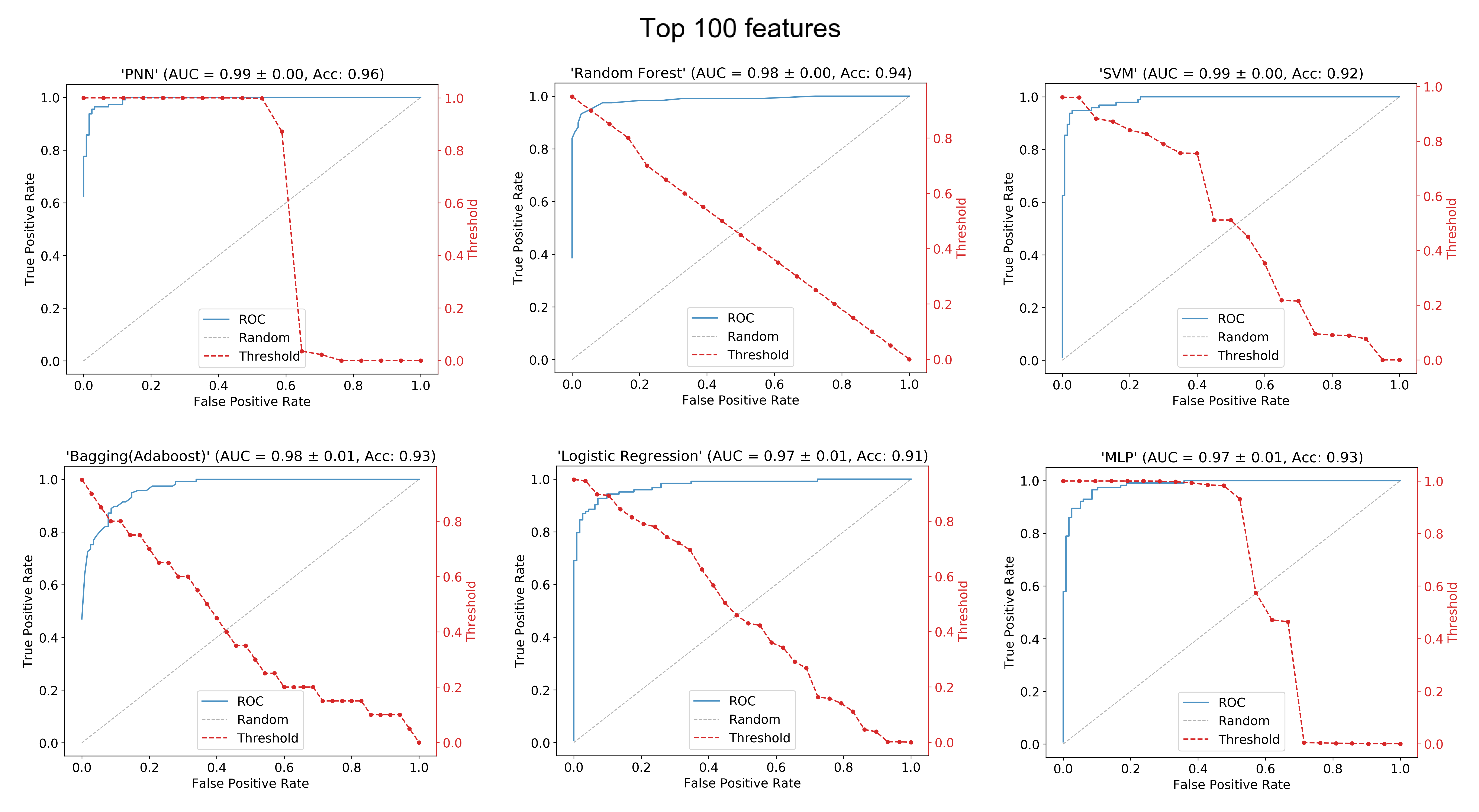


Figure S5: ROC curves of six ML classifiers overlaid with classification thresholds for all 696 (A) and top 100 (B) features. The thresholds are represented by red dots. This figure corresponds to Figure 6.

A.


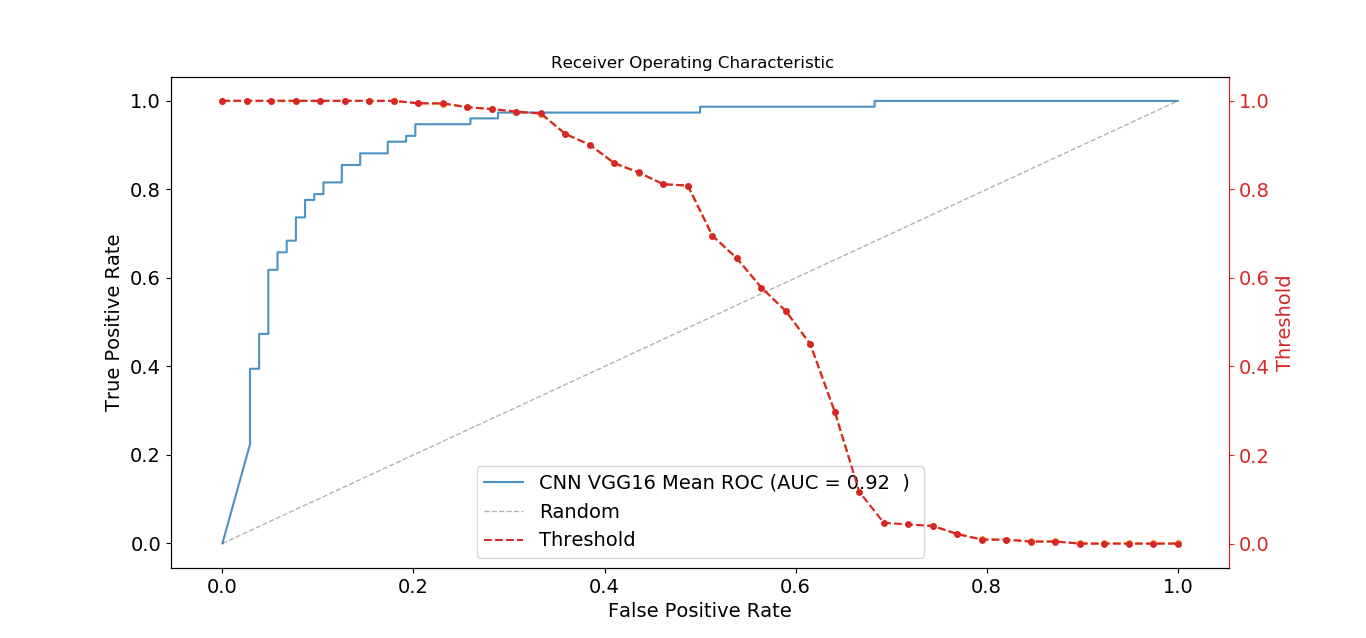


B.


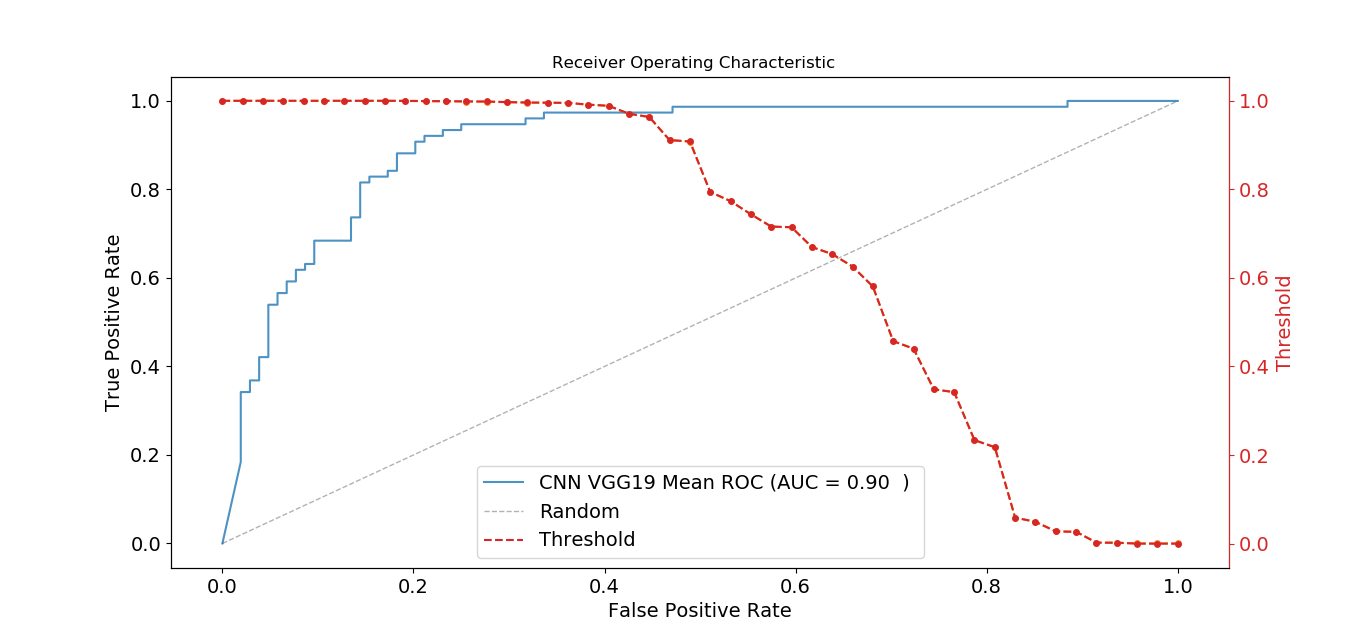


Figure S6: ROC curves overlaid with classification thresholds for CNN-based VGG16 (A) and VGG19 (B) models. The thresholds are represented by red dots. This figure corresponds to Figure 7.

A.


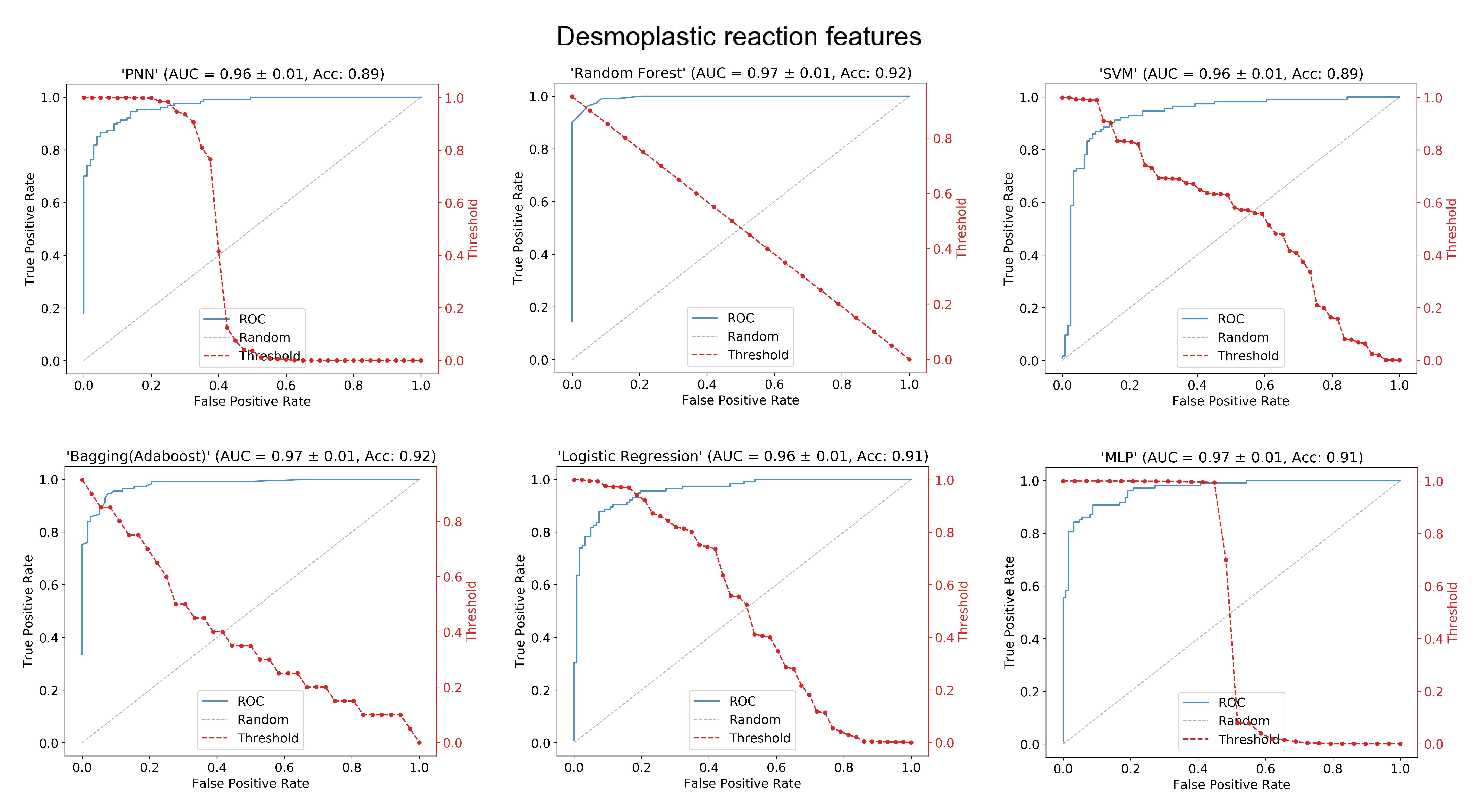


B.


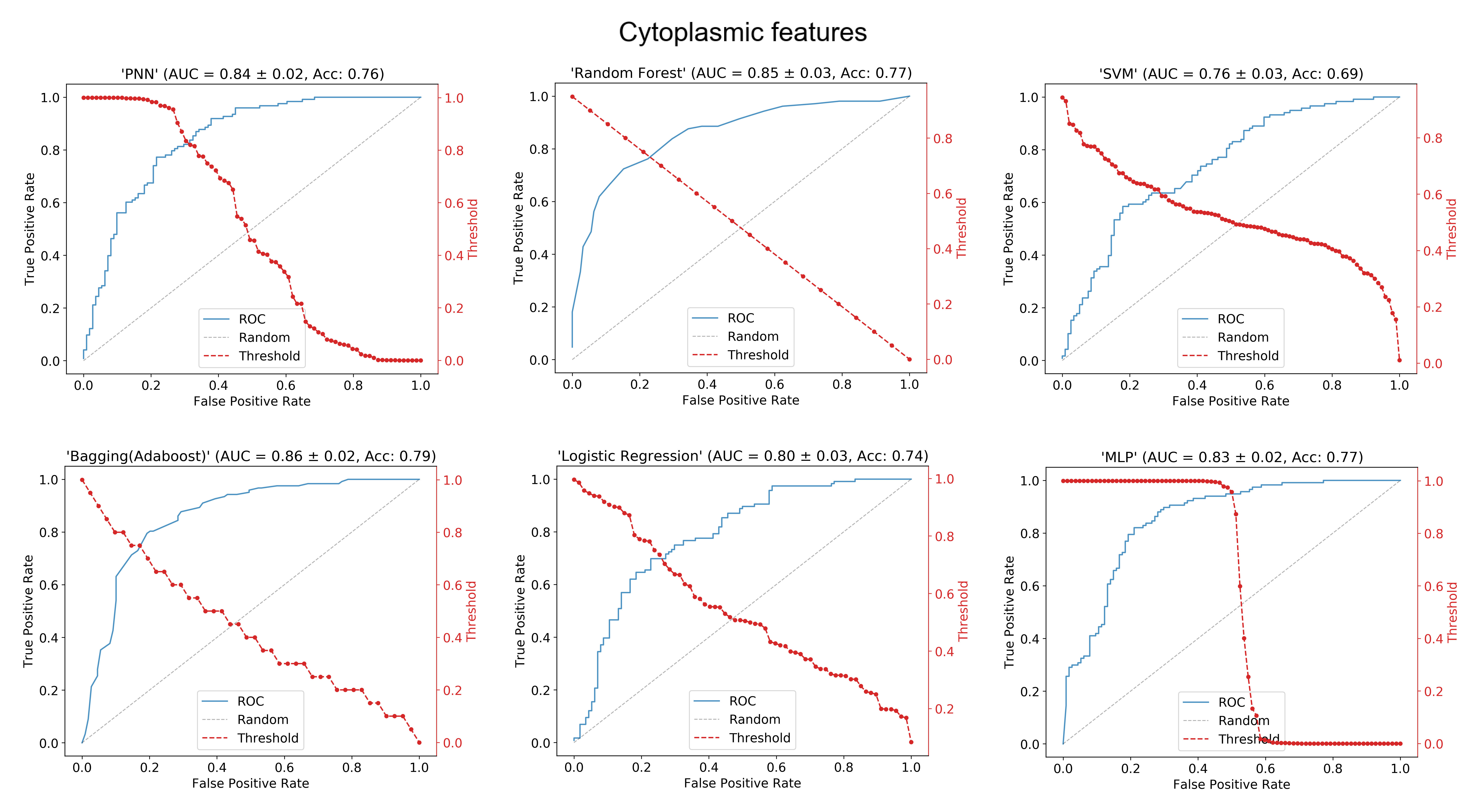


C.


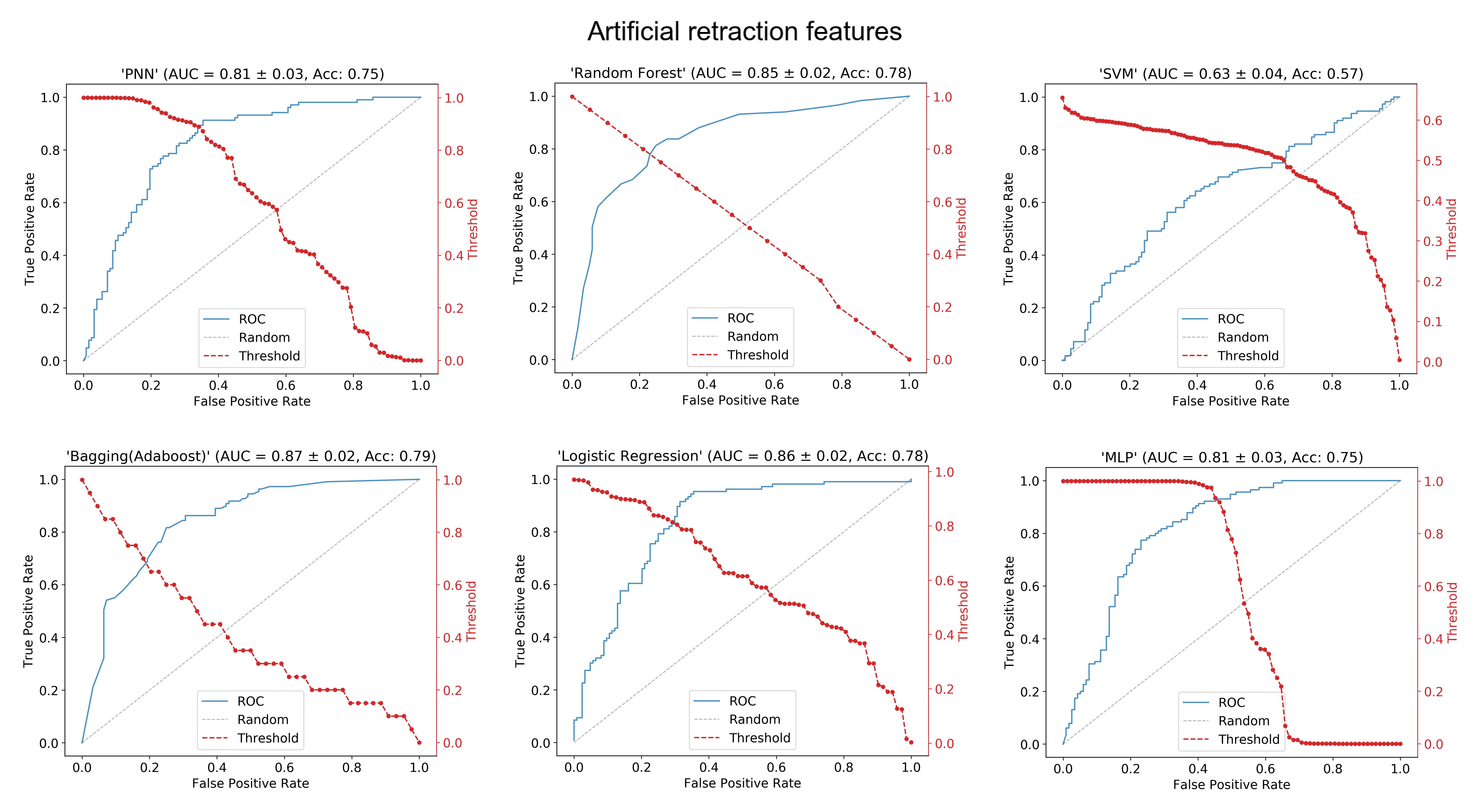


Figure S7: ROC curve overlaid with thresholds of various ML classifiers based on features related to desmoplastic reaction (A), cytoplasmic/eosin intensity (B), and retraction artifact (C). The thresholds are represented by red dots. This figure corresponds to Figure 8.

Figure S8: Ranked features from the desmoplastic reaction pattern. The ranking was based on the accumulated feature importance from bagged decision trees, in which the importance values were sorted from the highest to the lowest. The top 19 features are shown. The importance of a feature was computed as the (normalized) total reduction of the criterion brought by that feature. Ten features were related to nuclei (shown in blue) and nine features were related to connective tissues surrounding nuclei (shown in red).


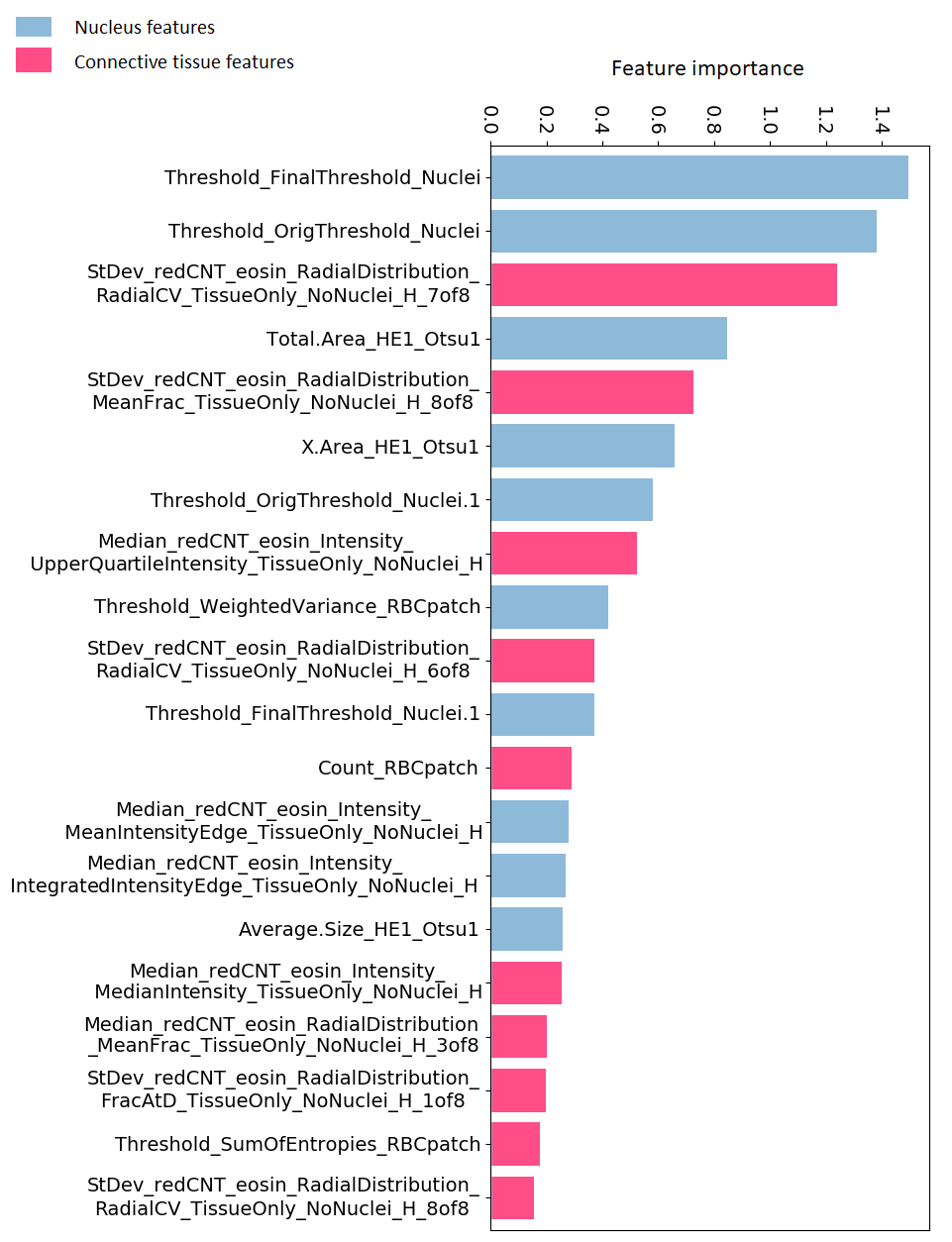

Supplement: Supplementary file 1 — Additional file 1. [file 12911_2020_1185_MOESM1_ESM.docx]
